# Supplementary material for: Diffusion MRI with free gradient waveforms on a high-performance gradient system: Probing restriction and exchange in the human brain
Source: ArXiv. 2023 Apr 5:arXiv:2304.02764v1. Preprint. [Version 1] (PMC10104199)
Supplement: Supplement 1 [file NIHPP2304.02764v1-supplement-1.pdf]

Supplementary material

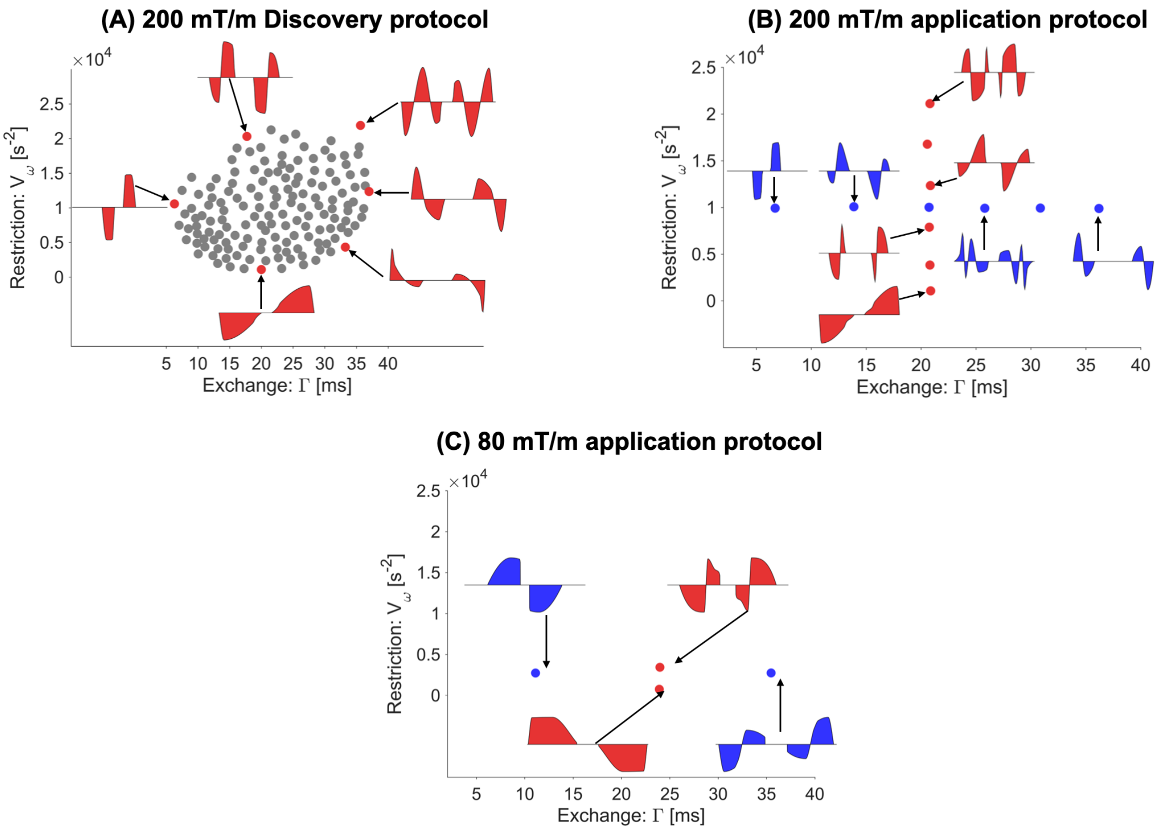

Figure A1: Protocols used for in vivo studies. (A) shows the 200 mT/m discovery protocol with a few waveform examples highlighted along the hull. (B) shows waveforms and restriction-exchange weightings of the 200 mT/m application protocol. (C) shows waveforms and restriction-exchange weightings for the 80 mT/m application protocol.

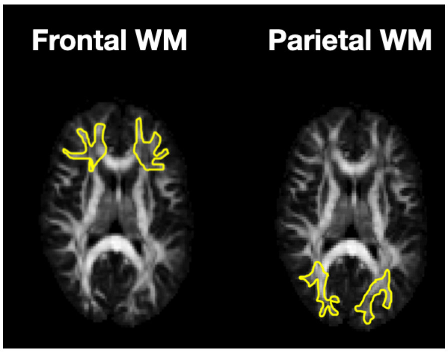

Figure A2: ROI placement in the frontal and parietal white matter.
